# Effects of Multi-Generational Rearing on Job’s Tears on the Performance and Host Plant Preference of *Spodoptera frugiperda* (Lepidoptera: Noctuidae)

**DOI:** 10.3390/insects16080773

**Published:** 2025-07-28

**Authors:** Feng-Luan Yao, Yao-Yao Wu, Gao-Ke Lei, Xiao-Yan Huang, Xue-Ling Ding, Xue-Song Lu, Yu Zheng, Yu-Xian He

**Affiliations:** 1 Institute of Plant Protection, Fujian Academy of Agricultural Sciences, Fuzhou 350013, China; wyydyxsjz@163.com (Y.-Y.W.); faaslgk@163.com (G.-K.L.); xue95711@163.com (X.-L.D.); m13509367296@163.com (X.-S.L.); zy848250@126.com (Y.Z.); 2 Fujian Key Laboratory for Monitoring and Integrated Management of Crop Pests, Fuzhou 350013, China; 3 Fujian Engineering Research Center for Green Pest Management, Fuzhou 350013, China; 4 Provincial Station of Plant Protection and Quarantine, Fujian Provincial Department of Agriculture, Fuzhou 350001, China; birdoftop@126.com

**Keywords:** fall armyworm, *Zea mays*, *Coix lacryma-jobi*, acclimation, host plant adaptation, life table, population projection

## Abstract

The fall armyworm (FAW), *Spodoptera frugiperda*, is a highly polyphagous pest posing a major threat to Poaceae crops, particularly corn (*Zea mays*), in China. However, its ability to adapt to Job’s tears (*Coix lacryma-jobi*), an edible and medicinal Poaceae species, under continuous rearing remains insufficiently understood. In this study, FAW survival, development, and reproduction were assessed over eight generations on two cultivars of Job’s tears, ‘Cuiyi 1’ and ‘Puyi 6’. Feeding and oviposition preferences were also examined in FAW populations reared on ‘Puyi 6’ by offering corn and ‘Puyi 6’ as hosts. Sustained rearing for five to eight generations on both cultivars significantly increased population fitness, with greater improvements observed in the ‘Puyi 6’ group. FAW reared on corn or ‘Puyi 6’ for two generations exhibited strong preferences for corn, whereas those reared on ‘Puyi 6’ for five to eight generations showed no significant host preference. These findings suggest that transgenerational adaptation markedly improved FAW performance and acceptance of Job’s tears, underscoring the need for intensified monitoring of FAW dynamics during the cultivation of Job’s tears.

## Simple Summary

Fall armyworm (FAW) is a destructive agricultural pest that can severely threaten cereal crops, such as corn. However, its adaptive response to Job’s tears, a nutritionally rich and medicinally valuable grain closely related to corn, remains poorly understood. In this study, FAW survival, development, and reproduction were explored on two varieties of Job’s tears (‘Cuiyi 1’ and ‘Puyi 6’), with assessments of feeding and oviposition preferences between corn and Job’s tears. FAW successfully completed its life cycle on both varieties. Following 5–8 generations of rearing on Job’s tears, the population performance improved, particularly on ‘Puyi 6’. Although early generations (e.g., the 2nd generation) exhibited strong preferences for corn in both feeding and oviposition, later generations (5th–8th), especially those reared on ‘Puyi 6’, demonstrated no distinct host preference. These findings indicate that Job’s tears may evolve into a high-risk host for FAW, with the potential for intensified infestations over successive generations, necessitating enhanced field surveillance by growers and researchers.

## 1. Introduction

Fall armyworm (FAW), *Spodoptera frugiperda* (J.E. Smith) (Lepidoptera: Noctuidae), is a major agricultural pest in tropical and subtropical regions [1]. Native to the Americas, FAW has invaded over 70 countries due to its strong migratory capacity, including most nations in Africa and Asia. Initial reports in Africa emerged in 2016 from countries such as Nigeria, Bénin, and Togo, followed by India in 2018 [2,3]. In China, FAW was first detected in Yunnan Province in December 2018 and has spread nationwide within a year [4,5].

FAW is highly polyphagous, infesting 353 plant species across 76 families, with Poaceae comprising the largest group at 30% (106 species) [6]. Poaceae crops such as corn, sorghum, rice, and various grasses serve as the suitable hosts for FAW, which comprises two host strains: the R-strain and C-strain [7]. Corn and sorghum are members of the Panicoideae subfamily and are favored by the C-strain, which have been recognized as the primary hosts of FAW invasions in Africa, Asia, and Oceania over the past decade [8,9,10,11]. In China, FAW field populations predominantly consist of inter-strain hybrids, with the RC hybrid being the most prevalent. This hybrid exhibits a genetic background largely derived from the C-strain with minor R-strain genomic components [12]. Field and laboratory studies have documented FAW infestations in at least 23 crops, 17 weeds, and three forage grasses; of these, 34.8% of crops, 64.7% of weeds, and all forage grasses belong to Poaceae [13,14,15,16,17,18]. These findings highlight the elevated vulnerability of Poaceae species to FAW, particularly under continued adaptive evolution following invasion.

Job’s tears, *Coix lacryma*-*jobi* L., is a member of the Panicoideae subfamily within the Poaceae family and a valuable plant resource with both edible and medicinal properties [19,20]. It is widely cultivated in tropical and subtropical regions and has garnered increasing attention in Asian countries as a functional food owing to its documented health benefits, including anti-cancer, anti-inflammatory, and antioxidant effects [20,21]. In China, Job’s tears is cultivated in at least 24 provinces, including Fujian, Guizhou, and Yunnan [22,23]. In Fujian Province, the cultivation spans approximately 6.7 × 10^3^ hm^2^, mainly in Pucheng County (Nanping City), Ninghua County (Sanming City), and Xianyou County (Putian City) [24]. The products from Pucheng and Ninghua were awarded China National Geographical Indication certification in 2008 and 2014, respectively. Following the FAW invasion into China, infestations were reported on 99 hm^2^ of Job’s tears in Guizhou and Hubei Provinces in 2019 [5]. In the same year, FAW presence was also observed in Fujian, with the affected area expanding to approximately 470 hm^2^ by 2020, posing a substantial threat to the production of Job’s tears.

Limited information is available regarding the performance of FAW on Job’s tears leaves. FAW can complete its life cycle on leaves, exhibiting comparable performance to that observed on sorghum, although lower than that of corn at both 25 °C and 30 °C [25]. In oviposition preference tests, FAW favors corn over Job’s tears and rarely selects buckwheat or kidney bean plants [26]. The feeding preferences of the 3rd and 4th instar larvae follow the same pattern, whereas the 5th instar larvae present no significant feeding bias between corn and Job’s tears [26]. Given that FAW infestations on Job’s tears have been documented in the field since 2019, its performance on this host may have improved, as continuous transgenerational rearing is known to facilitate host adaptation in herbivorous insects, including FAW [27,28,29]. For instance, FAW reared on two peanut varieties for eight generations exhibited significantly enhanced fitness [29], and the populations reared on rice for three generations developed increased feeding preferences, rendering rice leaves equally preferred to corn [28]. Nevertheless, no studies have investigated FAW adaptation to Job’s tears following the successive generations of rearing, despite certain field observations since 2019, suggesting the possibility of such evolutionary acclimation.

Therefore, this study aimed to assess the effects of continuous transgenerational rearing on Job’s tears leaves over eight generations on FAW performance, as well as its feeding and oviposition preferences. These findings could provide insights into the potential risk posed by FAW to Poaceae crops beyond corn, following prolonged invasion.

## 2. Materials and Methods

### 2.1. Plants

Three plant species were used in this study: one sweet corn cultivar, *Zea mays* L. var. ‘Jinyutian 1’ (Fuzhou Yongrong Seed Co., Ltd., Fuzhou City, China), and two Job’s tears cultivars, *Coix lacryma-jobi* var. ‘Cuiyi 1’ (Agricultural Sciences Institute of Ninghua County, Sanming City, China) and ‘Puyi 6’ (Agricultural Sciences Institute of Pucheng County, Nanping City, China), both widely cultivated and well known in China. Plants were grown in plastic pots (14.0 cm diameter × 13.5 cm high) filled with a growth medium consisting of a 1:1 (*v*/*v*) mixture of corn field soil and peat (Pindstrup Seeding, Pindstrup Mosebrug A/S, Pindstrup, Denmark) and 1 g slow-release fertilizer (17N-17P-17K, Osmocote 301, Wuhan Zhongken Fertilizer Co., Ltd., Wuhan City, China). All plants were cultivated pesticide-free in an air-conditioned greenhouse (25–28 °C) at the Fujian Academy of Agricultural Sciences (26°13′ N, 119°34′ E) under a natural photoperiod (12–15 h light and 12–9 h dark). The leaves were harvested for insect rearing, whereas the entire plant was utilized for the preference assays at the seedling stage (15–25 days after sowing). At that point, corn had 7–9 leaves, Job’s tears had 4–6 leaves, and the plant height ranged from 25 to 35 cm.

### 2.2. Insects

Approximately 300 third-to-fifth-instar FAW larvae were collected from a sweet corn field in Jian’ou County, Nanping City (26°38′ N, 117°58′ E), in October 2020. To establish a laboratory colony, larvae were provided with fresh leaves of the ‘Jinyutian 1’ sweet corn cultivar daily. These individuals were designated as the F0 generation and reared according to the protocol described by Yao et al. [29]. The insects were maintained in incubators at 26 ± 1 °C, 60 ± 5% relative humidity, and a 14:10 h light/dark photoperiod.

### 2.3. Effects of Multi-Generational Rearing on Job’s Tears Leaves on the Performance of FAW

Each FAW population reared on fresh plant leaves consisted of at least 15 mating pairs per generation. Eggs laid between 20:00 and 08:00 on the second and third days by no fewer than ten adult pairs were collected for subsequent rearing. The FAW larvae fed on corn leaves (CoJ) were reared up to the F2 generation, whereas those fed on Job’s tears cultivars ‘Cuiyi 1’ (JtC) and ‘Puyi 6’ (JtP) were reared to the F8 generation. From the F2 generation onward, a minimum of 450 eggs divided into six groups were used for transgenerational rearing. In the F2, F5, and F8 generations, at least 60 eggs were randomly selected from each group, and upon hatching, larvae were reared individually in Petri dishes (6.0 cm diameter, 1.5 cm height). Developmental progress was recorded daily, while the remaining eggs were reared in groups using transparent polypropylene containers (12.0 cm top diameter, 7.5 cm bottom diameter, 6.5 cm height). The adults were paired upon emergence and maintained individually. The oviposition and adult longevity were recorded daily until death. One-day-old 5th instar larvae and 2-day-old pupae from the F2 and F8 generations were weighed using an analytical balance (AX224ZH; OHAUS Instrument Co., Ltd., Shanghai City, China) with a precision of 0.0001 g. FAW typically pupated after the sixth-instar larvae, and any larvae exhibiting more than six instars were considered ‘supernumerary larvae’. Daily data were used to determine growth, reproductive performance, population parameters, and life table performance. The tests for the F2, F5, and F8 generations were conducted from 26 May to 22 July, 9 September to 15 October, and 11 December 2021, to 28 January 2022, respectively.

### 2.4. Effects of Multi-Generational Rearing on Job’s Tears Leaves on the Host Plant Preference of FAW

Leaf feeding and plant oviposition preferences were assessed using bi-choice assays, where one corn leaf (or plant) and one Job’s tears cultivar ‘Puyi 6’ leaf (or plant) were simultaneously offered. For the leaf feeding test, the leaves were cut into 1.5 cm diameter circular discs. Three discs from each plant species were arranged in a plastic Petri dish (9.0 cm diameter, 1.5 cm height), with approximately 2.0 cm between the discs of the same species and 3.5 cm between the discs of different species. Moistened filter paper was placed at the bottom of each dish. One 4th instar larva (1-day-old) that had been starved for 12 h and originated from the FAW populations reared on either corn or Job’s tears leaves was introduced into each dish. The dishes were sealed with perforated lids, and consumption was recorded after 6 h. Leaf discs were weighed before and after being provided to larvae. Water loss was determined by weighing the leaf discs initially and 6 h later in the absence of larvae. In cases where both corn and Job’s tears discs were consumed, the feeding preference was quantified as the proportion of weight loss for each plant species relative to the total weight loss per replicate. The test was replicated 30 times for the F2 generation of CoJ and JtP and 35 and 20 times for the F5 and F8 generations of JtP, respectively.

For oviposition preference tests, one corn plant and one Job’s tears plant were placed diagonally in opposite corners of a mesh cage (50 cm × 40 cm × 48 cm). A 3-day-old mated female, paired immediately after emergence, was introduced into each cage. Egg batches were counted 24 h after introduction. Oviposition preference was quantified as the percentage of egg batches laid on each plant relative to the total per replicate. The experiment was replicated 12 times for the F2 generation of CoJ and for the F2, F5, and F8 generations of JtP.

### 2.5. Life Table Data Analysis

Based on the age-stage, two-sex life table theory [30,31,32,33,34], the effects of continuous transgenerational rearing on Job’s tears leaves were analyzed in terms of FAW performance, including growth, reproductive metrics, life table parameters, and population parameters, using the TWOSEX-MSChart program (version 4/2/2025) [35,36,37]. The life table and population parameters included age-stage specific survival rate (*S*_xj_), age-specific survival rate (*l*_x_), female age-stage specific fecundity (*f*_x_), age-specific fecundity (*m*_x_), net maternity (*l*_x_*m*_x_), life expectancy (*e*_xj_), reproductive value (*v*_xj_), net reproductive rate (*R*_0_), intrinsic rate of increase (*r*), finite rate of increase (*λ*), and mean generation time (*T*). The definitions and equations of all parameters are provided in the Supplementary Materials.

### 2.6. Population Projection

The population growth of the F2 generation of CoJ and the F2, F5, and F8 generations of JtP over a 120-day period was simulated using the TIMING-MSChart program (version 5/7/2024) based on life table data [35]. The total population size at time *t* is denoted as *N*(*t*), which is calculated as the sum of all individuals at that time point:

$$N(t)=\sum_{j=1}^{\beta }\sum_{x=0}^{\infty }n_{xj,t}$$

where *n_xj,t_* is the number of individuals of age *x* and stage *j* at time *t* [38,39].

### 2.7. Statistical Analysis

For the performance parameters, the means and standard errors were obtained using the bootstrap method with 100,000 resamples, and treatment differences were assessed using the paired bootstrap test [40]. Differences in the projected population sizes were determined based on overlaps in the 95% confidence intervals. All analyses were conducted using the TWOSEX-MSChart program [35].

The effects of continuous rearing on the body weight of 5th instar larvae and pupae were analyzed using a generalized linear model (GLM) with gamma distribution and two-way ANOVA, respectively. Two-way ANOVA was applied when the assumptions of normality and homogeneity of variance were met by model residuals. Post hoc multiple comparisons were conducted using Tukey’s test. Feeding and oviposition preferences in bi-choice assays were analyzed using the Quade test because the data did not satisfy the assumptions required for Hotelling’s *T*^2^ test [41,42]. All statistical analyses were performed in R (version 4.3.3) [43]. The glm and aov functions from the base R package were used for GLM and ANOVA, respectively, whereas the quade.test function was used for preference analysis. Multiple comparisons were conducted using the emmeans function of the emmeans package. Statistical significance was set at *α* = 0.05.

## 3. Results

### 3.1. Effects of Multi-Generational Rearing on the Growth and Reproductive Performance of FAW

The pre-adult development time of FAW significantly declined from the F2 to the F8 generation when reared on the leaves of Job’s tears cultivar ‘Cuyi 1’, primarily due to a reduction in larval development duration (Table 1). This shortened larval period in later generations was associated with a decreased proportion of supernumerary larvae (61%, 30%, and 20% in the F2, F5, and F8 generations, respectively) and shorter duration of the 1st instar stage. Total pre-oviposition period (TPOP) was also significantly reduced in the F5 and F8 generations compared with that in the F2 generation. However, no significant differences were observed across generations in female adult longevity, total longevity, or the proportion of female adults (*N_f_*/*N*) and male adults (*N_m_*/*N*). Male adult longevity, the proportion of fertile female adults (*N_fr_*/*N_f_*), adult pre-oviposition period (APOP), oviposition day, and fecundity did not significantly differ between the F2 and F8 generations but were significantly reduced in the F5 generation compared to the F2 generation (Table 1). Nonetheless, compared with the F2 generation reared on corn leaves (CoJ), FAW reared on the leaves of Job’s tears cultivar ‘Cuyi 1’ (JtC) for eight generations exhibited a significantly longer pre-adult development time (owing to the extended larval duration), APOP, and TPOP, along with a markedly shorter oviposition period and substantially reduced fecundity (Table 1).

Similarly, the continuous rearing of FAW on the leaves of Job’s tears cultivar ‘Puyi 6’ resulted in a significantly shorter pre-adult development time from the F2 to the F8 generation, primarily due to reduced larval development duration (Table 2). The shortened larval period in later generations was associated with decreased duration across all larval instars. The total longevity and proportion of fertile female adults (*N_fr_*/*N_f_*) and male adults (*N_m_*/*N*) were not significantly affected by continuous rearing (Table 2). In contrast, female and male adult longevity, APOP, and TPOP were significantly reduced in the F5 and F8 generations compared with those in the F2 generation. The proportion of female adults (*N_f_*/*N*) and fecundity were significantly higher in the F8 generation than in the F2 and F5 generations (Table 2). However, when compared with the F2 generation reared on corn leaves (CoJ), FAW reared on the leaves of Job’s tears cultivar ‘Puyi 6’ (JtP) for eight generations exhibited a significantly longer pre-adult development time (due to the extended larval duration) and TPOP, as well as a markedly shorter total longevity and oviposition duration, along with substantially lower fecundity and proportion of fertile female adults (Table 2).

**Figure 1:**
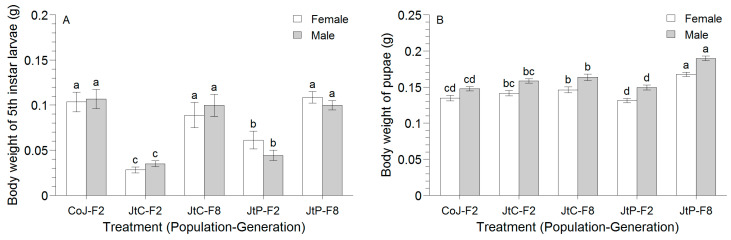
Effects of continuous rearing on the body weight of 5th instar larvae (**A**) and pupae (**B**) of FAW fed on corn for two generations (CoJ-F2) and continuously reared on Job’s tears cultivars ‘Puyi 6’ for two (JtP-F2) and eight generations (JtP-F8). Different letters above the bar indicate significant differences between treatments for female or male individuals (generalized linear model with gamma distribution and two-way ANOVA followed by Tukey’s test).

The body weights of the 5th instar larvae (χ^2^ = 165.8, *df* = 4, *p* < 0.001) and pupae (*F* = 37.1, *df* = 4, 279, *p* < 0.001) differed significantly among treatments (Figure 1). The larval body weight significantly increased from the F2 to F8 generation in FAW reared on Job’s tears leaves, resulting in no significant difference between the F8 generations of JtC and JtP and the F2 generation of CoJ (Figure 1A). In contrast, a significant increase in the body weight of pupae following continuous rearing was observed only in JtP (Figure 1B). The body weight of pupae in the F2 generation of CoJ was comparable to that in the F2 generation of JtC and JtP but was substantially lower than that in the F8 generation. No significant sex-based difference was observed in the body weight of the 5th instar larvae (χ^2^ = 0.03, *df* = 1, *p* = 0.871; Figure 1A), whereas the body weight of male pupae was significantly higher than that of female pupae across all treatments (*F* = 62.6, *df* = 1, 279, *p* < 0.001; Figure 1B). No significant interaction was identified between the treatments and genders for the body weights of either larvae (χ^2^ = 7.1, *df* = 4, *p* = 0.132) or pupae (*F* = 0.4, *df* = 4, 279, *p* = 0.799).

### 3.2. Effects of Continuous Rearing on the Life Table Performance of FAW

**Figure 2:**
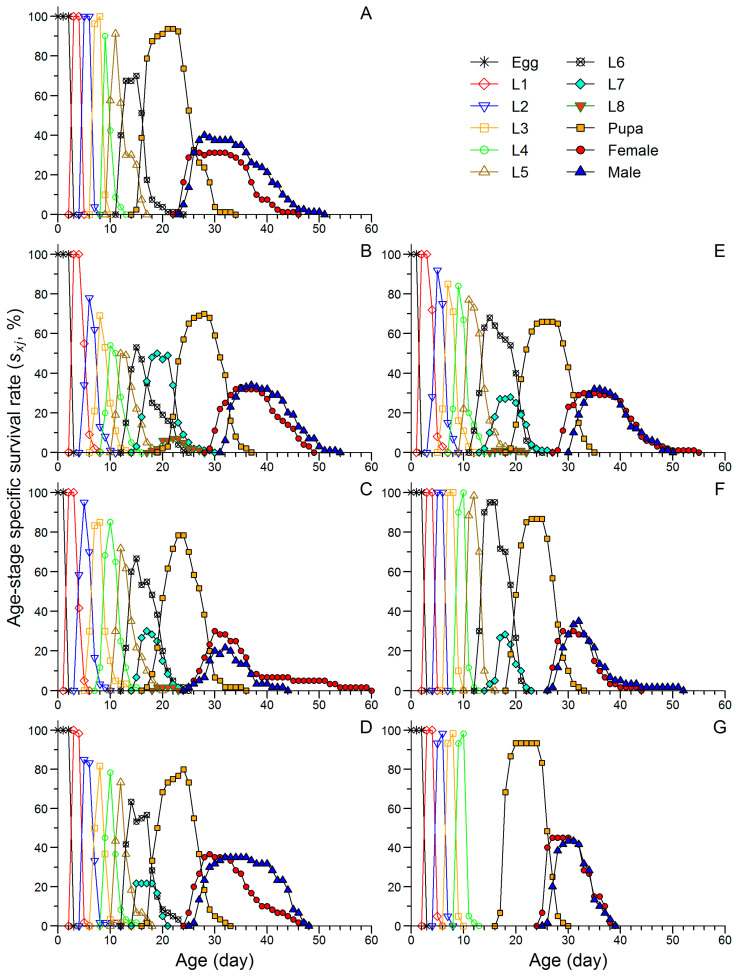
Age-stage specific survival rate (*s_xj_*) of FAW fed on corn for two generations (**A**) and continuously reared on Job’s tears cultivars ‘Cuiyi 1’ (**B**–**D**) and ‘Puyi 6’ (**E**–**G**) for eight generations. Panels represent different generations: F2 (**B**,**E**), F5 (**C**,**F**), and F8 (**D**,**G**).

Figure 2 illustrates the stage overlap in FAW populations reared on corn and Job’s tears leaves. In general, the peak survival rates at each developmental stage were higher in the F2 generation of CoJ than in the F2, F5, and F8 generations of JtC, and were comparable to those in the F5 and F8 generations of JtP. The larval and pre-adult survival rates in JtC did not change significantly across generations but remained significantly lower than those observed in the F2 generation of CoJ (Table S1). In contrast, JtP exhibited significant increases in the larval and pre-adult survival rates in the F5 or F8 generations, resulting in larval survival comparable to that of CoJ and pre-adult survival significantly exceeding that of CoJ (Table S2).

**Figure 3:**
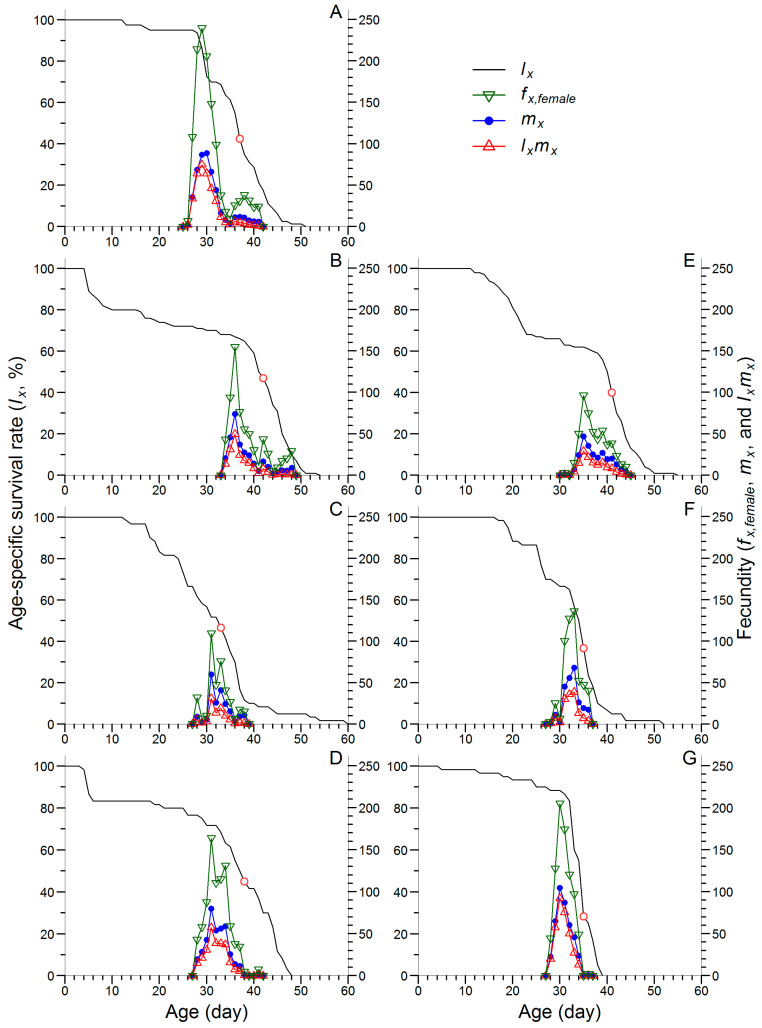
Age-specific survival rate (*l_x_*), female age-stage specific fecundity (*f_x,female_*), and age-specific fecundity (*m_x_*) and net maternity (*l_x_m_x_*) of FAW reared on corn for two generations (**A**) and continuously reared on Job’s tears cultivars ‘Cuiyi 1’ (**B**–**D**) and ‘Puyi 6’ (**E**–**G**) for eight generations. Panels represent different generations: F2 (**B**,**E**), F5 (**C**,**F**), and F8 (**D**,**G**). The points in the *l*_x_ curves indicate the age (in days) at which the survival rate drop below 50%: days 37, 42, 33, 38, 41, 35, and 35 in panels (**A**–**G**), respectively.

Figure 3 shows that the age-specific survival rate (*l*_x_) declined rapidly within the first 5 d in the F2 and F8 generations of JtC, indicating a higher mortality rate of the first instar larvae in these two generations than in the F5 generation (Figure 3B–D and Table S1). In contrast, the *l*_x_ curve of the F8 generation of JtP declined gradually over the first 30 d, reflecting a lower mortality rate than that observed in the F2 and F5 generations of JtP (Figure 3E–G). The *l*_x_ value dropped below 50% (and reached zero) on days 37 (51), 42 (54), 33 (60), 38 (48), 41 (55), 35 (52), and 35 (39) in the F2 generation of CoJ and in the F2, F5, and F8 generations of JtC and JtP, respectively (Figure 3).

Figure 3 also shows that the peak values of female age-stage specific fecundity (*f_x,female_*), age-specific fecundity (*m*_x_), and net maternity (*l*_x_*m*_x_) were identified on day 29 in the F2 generation of CoJ, which was several days earlier than those in the F2, F5, and F8 generations of JtC (days 36, 31, and 31, respectively) and JtP (days 35, 33, and 31, respectively). These peak values generally increased from the F2 to F8 generations in both JtC and JtP. The highest *f_x,female_* value was recorded in the F2 generation of CoJ (239.6 eggs), whereas the maximum *m*_x_ and *l*_x_*m*_x_ values were observed in the F8 generation of JtP, reaching 87.2 and 75.6 eggs, respectively.

**Figure 4:**
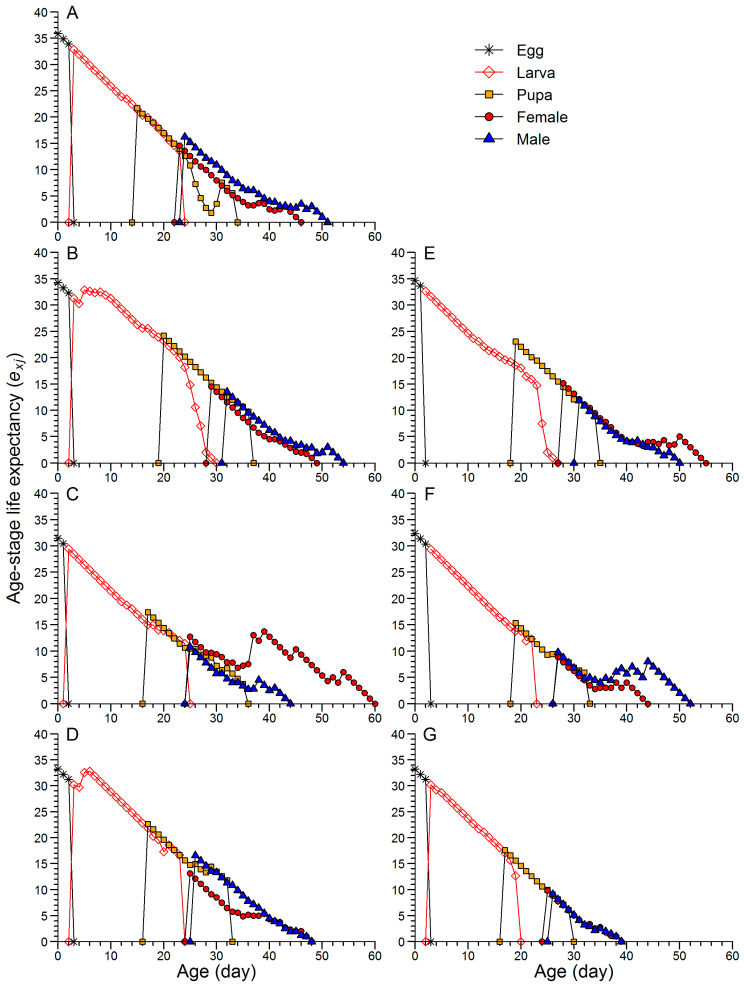
Age-stage life expectancy (*e_xj_*) of FAW reared on corn for two generations (**A**) and continuously reared on Job’s tears cultivars ‘Cuiyi 1’ (**B**–**D**) and ‘Puyi 6’ (**E**–**G**) for eight generations. Panels represent different generations: F2 (**B**,**E**), F5 (**C**,**F**), and F8 (**D**,**G**).

The age-stage life expectancy (*e_xj_*) of a newly hatched egg in the F2 generation of CoJ was 35.9 d, which was higher than that observed in the F2, F5 and F8 generations of JtC (34.3, 31.4, and 33.2 d, respectively) and JtP (34.7, 32.4, and 33.2 d, respectively) (Figure 4). The highest *e_xj_* for female and male adults was recorded in the F2 generation of JtP (15.1 d) and F8 generation of JtC (16.6 d), respectively (Figure 4). In JtP, the *e_xj_* of female and male adults declined substantially in the F5 (8.9 and 9.8 d) and F8 (9.8 and 9.1 d) generations compared with the F2 generation (15.1 and 11.8 d).

**Figure 5:**
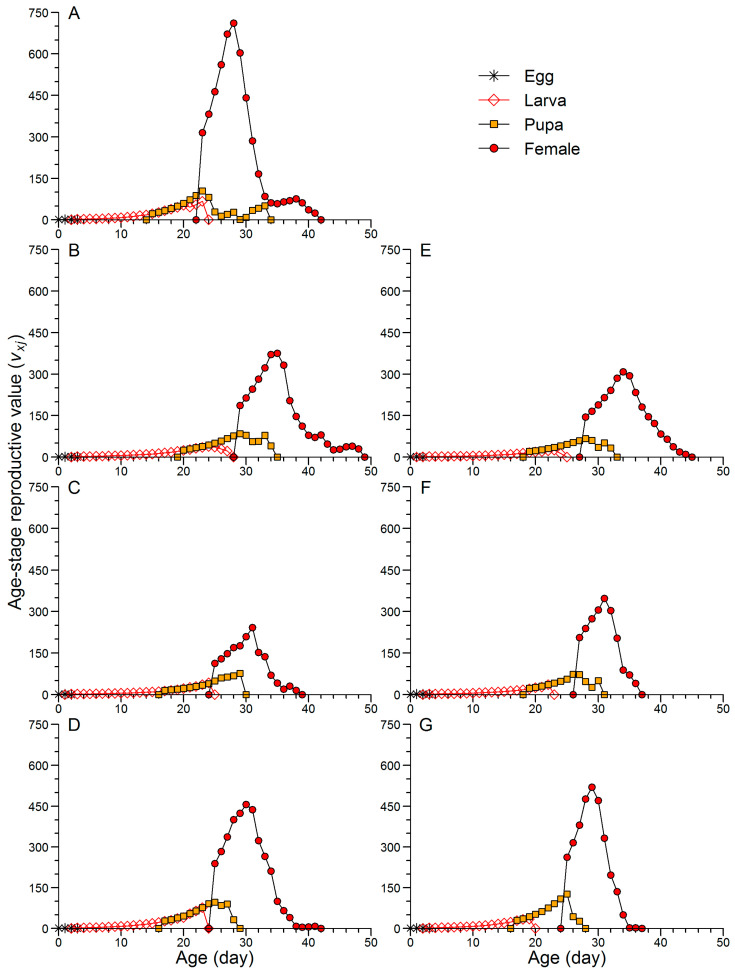
Age-stage reproductive value (*v*_xj_) of FAW reared on corn for two generations (**A**) and continuously reared on Job’s tears cultivars ‘Cuiyi 1’ (**B**–**D**) and ‘Puyi 6’ (**E**–**G**) for eight generations. Panels represent different generations: F2 (**B**,**E**), F5 (**C**,**F**), and F8 (**D**,**G**).

Figure 5 illustrates that, in the F2 generation of CoJ, female adults emerged on day 23, with an age-stage reproductive value (*v*_xj_) of 315.3. In the JtC strain, the corresponding values were 186.5 (F2, day 29), 112.5 (F5, day 25), and 239.2 (F8, day 25), while in JtP, the values were 144.9 (F2, day 28), 205.7 (F5, day 27), and 263.0 (F8, day 25). The peak values in the F2, F5, and F8 generations of JtC were 375.2 (day 35), 242.2 (day 31), and 455.7 (day 30), respectively. The corresponding peaks in JtP were 308.3 (day 34), 346.9 (day 31), and 519.0 (day 29). Although the continuous rearing on Job’s tears leaves led to increased *v*_xj_ values in later generations, the highest peak reproductive value was still observed in the F2 generation of CoJ, reaching 771.9 on day 28.

### 3.3. Effects of Continuous Rearing on the Population Parameters of FAW

Continuous rearing on the leaves of both Job’s tears cultivars substantially increased the intrinsic rate of increase (*r*) and finite rate of increase (*λ*), while significantly reducing the mean generation time (*T*) (Table 3 and Table 4). Compared with the F2 generation of CoJ, the F8 generations of JtC and JtP exhibited comparable net reproductive rates (*R*_0_) but longer *T* values. Notably, the F8 generation of JtC demonstrated significantly lower *r* and *λ* than the F2 generation of CoJ, whereas the F8 generation of JtP exhibited similar *r* and *λ* values (Table 3 and Table 4).

### 3.4. Effects of Continuous Rearing on the Population Projection of FAW

**Figure 6:**
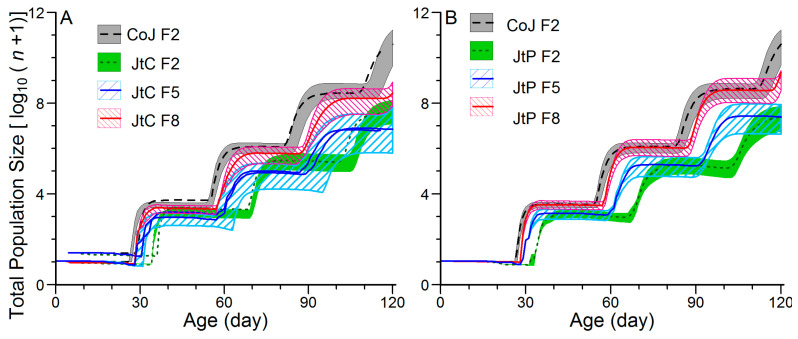
Total population size projection of FAW reared on corn for two generations (CoJ F2) and continuously reared on Job’s tears cultivars ‘Cuiyi 1’ (**A**) and ‘Puyi 6’ (**B**) for eight generations (JtC F2, F5, and F8; JtP F2, F5, and F8). Total population size is shown as the mean (line) ± 95% confidence interval (shaded area).

The population growth capacity was projected from ten newly hatched eggs over a 120-day period using life table data (Figure 6 and Figure S1). The stage-size projections indicated that the F2 generations of JtC and JtP completed two generations, whereas the F2 generation of CoJ and the F5 and F8 generations of JtC and JtP completed three, reflecting a faster developmental rate (Figure S1). The total population size projections showed the highest mean in the F2 generation of CoJ, followed by the F8 generation and the lowest values in the F2 and F5 generations of JtC and JtP. However, the 95% confidence intervals for the total population size overlapped between the F8 generation and F2 and F5 generations of JtC. In contrast, the 95% confidence interval in the F8 generation of JtP was generally higher than that in the F2 and F5 generations, suggesting that continuous rearing enhanced the population growth potential of JtP more effectively than JtC.

### 3.5. Effects of Continuous Rearing on Feeding and Oviposition Preference of FAW

**Figure 7:**
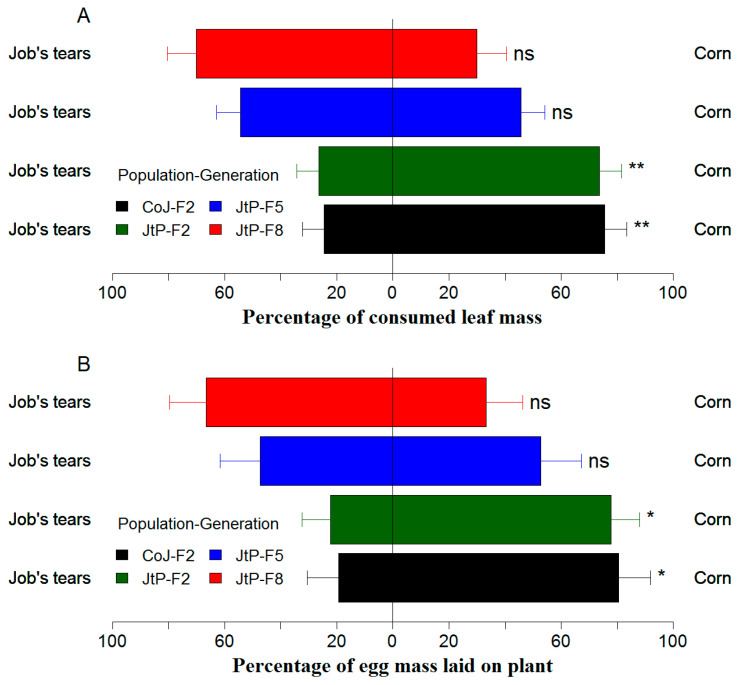
Effects of continuous rearing on feeding (**A**) and oviposition (**B**) preference of FAW reared on corn for two generations (CoJ–F2) and continuously reared on Job’s tears cultivar ‘Puyi 6’ for two, five, and eight generations (JtP–F2, –F5, and –F8). ns, *, and ** represent *p* > 0.05, <0.05, and <0.01, respectively (Quade test).

The F2 generation of CoJ and JtP exhibited a significant preference for feeding on corn leaves over Job’s tears leaves (Figure 7A; CoJ: *F* = 9.1, df = 1, 29, *p* = 0.005; JtP: *F* = 8.9, df = 1, 29, *p* = 0.006) and ovipositing on corn over Job’s tears (Figure 7B; CoJ: *F* = 6.9, df = 1, 11, *p* = 0.023; JtP: *F* = 7.3, df = 1, 11, *p* = 0.020). Conversely, the F5 and F8 generations of JtP presented no significant feeding preference (F5: *F* = 0.3, df = 1, 34, *p* = 0.619; F8: *F* = 3.6, df = 1, 19, *p* = 0.072) or oviposition preference (F5: *F* = 0.8, df = 1, 11, *p* = 0.809; F8: *F* = 1.7, df = 1, 11, *p* = 0.222) between the corn and Job’s tears (cultivar ‘Puyi 6’).

## 4. Discussion

Since its invasion of China, FAW has demonstrated remarkable adaptability to non-preferred host plants, posing a serious threat to Poaceae crops, such as Job’s tears, sugarcane, sorghum, and wheat, in addition to its primary host, corn [2,5,25,44,45,46,47,48]. Our findings revealed that FAW can complete its life cycle and sustain population growth when consecutively reared on Job’s tears leaves, highlighting the urgent need for enhanced monitoring and management of FAW infestations in this crop.

The beneficial effects of continuous and transgenerational rearing as well as host plant acclimation on insect performance have been well documented. For instance, the pea aphid *Acyrthosiphon pisum* (Harris) (Hemiptera: Aphididae) significantly improved sap-feeding efficiency after six months of acclimation to two novel host plants, facilitating the establishment of stable colonies [49]. Similarly, FAW reared on rice for 20 generations exhibited markedly reduced larval development time and increased larval and pupal survival rates compared with the first generation [50]. Both this study and previous research by our team [29] suggested that FAW reared on non-preferred host plants such as Job’s tears and peanut for five to eight generations significantly enhanced larval and adult performance, as well as overall population fitness. Furthermore, the effects of continuous rearing may differ between laboratory and wild populations. Coudron et al. [51] reported that the developmental time, pre-oviposition period, fecundity, and the 2nd instar nymphal survival exhibited the significant improvements in a laboratory colony but remained relatively unchanged in a wild colony when fed an artificial diet for eleven generations. In the present study, FAW subjected to continuous rearing beginning in May 2021 originated from a field population collected in October 2020, suggesting that the observed improvements more accurately reflected the adaptive responses of field-derived populations.

The positive effects of continuous rearing vary across host plant species. For example, more pronounced effects on the body mass and relative growth rate of the mustard leaf beetle *Phaedon cochleariae* (F.) (Coleoptera: Chrysomelidae) were observed on *Sinapis alba* than on *Nasturtium officinale* after ten generations of continuous rearing [27]. Similarly, the present study showed that after eight generations of rearing, FAW fed on the Job’s tears cultivar ‘Puyi 6’ exhibited more substantial improvements in the larval and pre-adult survival rates and in population parameters (*r* and *λ*) than those fed on ‘Cuiyi 1’ (Tables S1 and S2, Table 3, and Table 4). The value of *T* was also more significantly reduced in the ‘Puyi 6’ group (Table 3 and Table 4). Additionally, while the 5th instar larval weight increased significantly after the continuous rearing on both cultivars, the body weight of pupae increased only in the ‘Puyi 6’ group (Figure 1), indicating more pronounced positive effects on the individuals fed on ‘Puyi 6’ than on ‘Cuiyi 1’. In contrast, a previous study showed opposing effects in FAW fed on two rice cultivars, where larval body weight increased with *japonica* rice but decreased with *indica* rice after three generations of rearing [28].

In addition, the positive effects of continuous rearing may diminish over successive generations because of inbreeding depression. For instance, parasitoid quality has been shown to improve and peak during early generations but decline sharply under prolonged mass rearing conditions [52,53]. In this study, FAW reared on ‘Cuiyi 1’ exhibited a significant reduction in fecundity in the F5 generation (Table 1), while those reared on ‘Puyi 6’ showed the marked decreases in both the female and male adult longevity in the F5 and F8 generations (Table 2). These observations suggest a potential risk of inbreeding depression even during the relatively early stages of continuous rearing. However, inbreeding depression was not observed in FAW fed on rice or maize for 20 generations [50].

In the F2 generation of CoJ, as well as the F2 and F8 generations of JtC and JtP, female adults emerged 1–3 d earlier than males (Figure 2). A similar pattern was reported by Wang et al. [54], who observed earlier emergence of female adults than male adults in FAW populations fed on six cash crops and attributed this to the migratory behavior of the species. Specifically, early female emergence may facilitate improved access to food and greater energy accumulation, both of which are critical for survival and reproduction. However, in contrast to these findings, no sex-based difference in emergence timing was observed in the F5 generation of JtC and JtP, where females and males emerged simultaneously. In this study, adult emergence occurred in June, October, and January for the F2, F5, and F8 generations, respectively. We hypothesized that the timing of female versus male emergence could be influenced by certain seasonal factors, such as variations in temperature, humidity, and food availability, which may differentially affect developmental rates and emergence timing in FAW.

Characterized by age-stage-specific parameters, the life table performance of FAW clearly indicated that continuous rearing enhanced growth and reproductive capacity in later generations. This trend was supported by several observations. (1) In the F5 and F8 generations, *l*_x_ declined below 50% earlier than that in the F2 generation (Figure 3). (2) In the F8 generation of JtC and the F5 and F8 generations of JtP, the peak values of *f_x,female_*, *m*_x_ and *l*_x_*m*_x_ occurred earlier and were higher than those in the F2 generation (Figure 3). (3) The *e_xj_* representing the average longevity within a cohort was lower in the F5 and F8 generations than in the F2 generation (Figure 4). (4) The onset of reproduction and the day of maximum *v*_xj_ occurred earlier in the F5 and F8 generations, with the highest *v*_xj_ consistently observed in the F8 generation. Wang et al. [54] also reported that FAW reared on corn and wheat exhibited the earlier and higher peaks of *f_x,female_*, *m*_x_ and *l*_x_*m*_x_, as well as lower *e_xj_* than those reared on soybean, tomato, cotton, and Chinese cabbage. This is consistent with their shorter pre-adult duration and higher fecundity. In contrast, Jin et al. [55] found that, in the papaya mealybug *Paracoccus marginatus* Williams and Granara de Willink (Hemiptera: Pseudococcidae), earlier identification of a survival rate of less than 50%, earlier but lower peaks of *m*_x_ and *l*_x_*m*_x_, and reduced *e_xj_* were associated with reduced fecundity. Aligning with the findings of Wang et al. [54], Jin et al. [55], and Zhao et al. [56], this study suggested that the earlier emergence of female adults in the *v*_xj_ curves played a key role in elevating *r* and *λ*. Indeed, the first reproductive age and TPOP could be critical determinants of certain population growth parameters such as *r* and *λ* [37,55].

The capacity for population expansion can be effectively demonstrated using population size projections. Larger simulated population sizes and increased generational turnover are consistently associated with greater population fitness, providing a useful framework for evaluating the host plant suitability for insects [54,55,56]. In this study, the projected population size was consistently higher in the F2 generation of CoJ than in JtC and JtP (Figure 6). Moreover, the projections reflected the phenotypic plasticity of FAW during adaptation to less preferred host plants, with continuous rearing significantly enhancing adaptability. These findings also suggest a potential evolutionary response of FAW to local host plants, as phenotypic plasticity has been proposed to drive genetic adaptation over time [57]. Notably, the overlaps in the projected total population sizes between the F2 generation of CoJ and the F8 generations of JtC and JtP within a 120-day period suggested that, after eight generations of transgenerational adaptation, FAW could reach levels of abundance that may cause serious damage to Job’s tears.

Continuous rearing not only improves the FAW performance on non-preferred host plants but also alters its host preference toward those less suitable. In this study, the JtP populations initially exhibited a preference for corn but shifted to a non-biased selection between corn and Job’s tears after five to eight generations of adaptation (Figure 6). Similarly, FAW populations reared continuously for 15–20 generations on corn or rice exhibited a marked oviposition preference for the corresponding host [50]. However, FAW reared for three generations on two rice cultivars showed no significant change in feeding or oviposition preferences for corn [54]. Whether such preference shifts are generation-dependent and whether the five generations represent the minimum threshold for preference alteration require further research. The preference–performance hypothesis posits that adult oviposition decisions can be positively correlated with offspring performance [58]. Accordingly, the non-biased preference for leaves and plants between corn and Job’s tears demonstrated similar performance of the F5 and F8 generations of JtP on both host leaves. This implied that FAW adaptation to corn could be disrupted after five to eight generations of rearing on Job’s tears, as the JtP performance in the F5 and F8 generations remained inferior to that of CoJ in the F2 generation (Table 2 and Table 4; Figure 3 and Figure 5). On the other hand, the performance of *P. cochleariae* on its preferred host was unaffected after ten generations of acclimation to two alternative hosts [27]. Further validation is needed to clarify the link between host preference and performance in FAW populations acclimated to Job’s tears, when both corn and Job’s tears are available.

## 5. Conclusions

This study demonstrated that continuous rearing for five to eight generations significantly enhanced FAW adaptation, as well as feeding and oviposition preference, towards Job’s tears, highlighting the species’ plasticity in adapting to novel host plants, particularly Poaceae crops. This study was conducted under controlled laboratory conditions with constant temperature and humidity and fresh young leaves were used. However, FAW in the field experiences various environmental stresses, including temperature fluctuations and senescent leaves with lower nutritional quality. Therefore, the observed transgenerational adaptation requires further validation using field-based assessments of FAW survival, development, and fecundity. Additionally, as the FAW colony used in this study originated from corn fields, its performance on Job’s tears may differ from that of field populations already established on Job’s tears. Future studies should incorporate sampling from Job’s tears-infested fields to evaluate both preference and performance under natural conditions, which will provide a more accurate understanding of FAW occurrence and potential damage to Job’s tears in the coming years.

## Figures and Tables

**Table 1:** Effects of continuous rearing on the leaves of Job’s tears cultivar ‘Cuyi 1’ on the development and reproduction of FAW.

Parameters	Corn		Job’s Tears Cultivar ‘Cuiyi 1’					
	*n*	F2 Generation	*n*	F2 Generation	*n*	F5 Generation	*n*	F8 Generation
Development time (d)								
Pre-adult	59	25.5 ± 0.2 d	68	32.5 ± 0.2 a	36	28.8 ± 0.4 b	45	27.6 ± 0.3 c
Eggs	80	3.0 ± 0.0 a	100	3.0 ± 0.0 a	60	2.0 ± 0.0 b	60	3.0 ± 0.0 a
Larvae	77	14.0 ± 0.2 d	70	20.3 ± 0.2 a	49	18.5 ± 0.2 b	48	16.3 ± 0.2 c
1st instar	80	2.0 ± 0.0 c	85	2.7 ± 0.1 a	60	2.5 ± 0.1 b	51	2.0 ± 0.0 c
2nd instar	80	2.0 ± 0.0 b	82	2.3 ± 0.1 a	60	2.5 ± 0.1 a	50	2.5 ± 0.1 a
3rd instar	80	2.1 ± 0.0 c	81	2.2 ± 0.1 b	59	2.5 ± 0.1 a	50	2.1 ± 0.1 bc
4th instar	80	1.5 ± 0.1 c	80	2.2 ± 0.1 b	58	2.7 ± 0.1 a	50	2.1 ± 0.1 b
5th instar	78	3.0 ± 0.2 a	80	2.4 ± 0.1 b	57	2.5 ± 0.1 b	50	2.3 ± 0.1 b
6th instar	57	4.6 ± 0.1 a	78	3.6 ± 0.2 b	51	3.9 ± 0.2 b	49	4.0 ± 0.2 b
7th instar	/	/	54	5.4 ± 0.2 a	17	4.7 ± 0.4 a	12	4.9 ± 0.2 a
8th instar	/	/	7	5.6 ± 0.4 a	1	4.0 b	/	/
Pupae	59	8.6 ± 0.1 b	68	9.1 ± 0.1 a	36	8.6 ± 0.2 b	45	8.4 ± 0.1 b
Female adult longevity (d)	27	12.6 ± 0.7 a	33	12.2 ± 0.6 a	20	10.2 ± 1.6 a	22	11.5 ± 0.9 a
Male adult longevity (d)	32	14.4 ± 0.9 a	35	12.0 ± 0.7 b	16	6.6 ± 0.9 c	23	14.3 ± 0.9 ab
Total longevity (d)	80	35.9 ± 0.8 a	100	34.3 ± 1.6 ab	60	31.4 ± 1.2 b	60	33.2 ± 1.8 ab
Proportion of female adults (*N_f_*/*N*) (%)	80	33.8 ± 5.3 a	100	33.0 ±4.7 a	60	33.3 ± 6.1 a	60	36.7 ± 6.2 a
Proportion of fertile female adults (*N_fr_*/*N_f_*) (%)	80	96.3 ± 3.7 a	100	84.8 ± 6.3 a	60	50.0 ± 11.4 b	60	90.9 ± 6.2 a
Proportion of male adults (*N_m_*/*N*) (%)	80	40.0 ± 5.5 a	100	35.0 ± 4.8 a	60	26.7 ± 5.7 a	60	38.3 ± 6.3 a
APOP (d)	26	3.4 ± 0.2 b	28	5.2 ± 0.5 a	10	3.9 ± 0.5 b	20	5.1 ± 0.7 a
TPOP (d)	26	28.4 ± 0.4 c	28	36.6 ± 0.5 a	10	31.7 ± 0.7 b	20	31.6 ± 0.7 b
Oviposition day (*O_d_*) (d)	26	5.5 ± 0.2 a	28	4.2 ± 0.4 b	10	3.3 ± 0.3 c	20	4.1 ± 0.4 bc
Fecundity (eggs/female)	27	1054.6 ± 66.6 a	33	542.4 ± 69.8 b	20	278.1 ± 76.8 c	22	734.4 ± 109.0 b

Data are presented as mean ± SE. Different letters within each row indicate significant differences between treatments at the level of *α* = 0.05 (paired bootstrap test). APOP and TPOP refer to adult pre-oviposition period and total pre-oviposition period, respectively.

**Table 2:** Effects of continuous rearing on the leaves of Job’s tears cultivar ‘Puyi 6’ on the development and reproduction of FAW.

Parameters	Corn		Job’s Tears Cultivar ‘Puiyi 6’					
	*n*	F2 Generation	*n*	F2 Generation	*n*	F5 Generation	*n*	F8 Generation
Development time (d)								
Pre-adult	59	25.5 ± 0.2 d	63	31.3 ± 0.2 a	41	28.7 ± 0.2 b	54	26.6 ± 0.1 c
Eggs	80	3.0 ± 0.0 a	100	2.0 ± 0.0 b	60	3.0 ± 0.0 a	60	3.0 ± 0.0 a
Larvae	77	14.0 ± 0.2 d	67	19. 5 ± 0.2 a	52	17.4 ± 0.1 b	56	15.3 ± 0.1 c
1st instar	80	2.0 ± 0.0 b	100	2.8 ± 0.1 a	60	2.0 ± 0.0 b	59	2.0 ± 0.0 b
2nd instar	80	2.0 ± 0.0 b	100	2.2 ± 0.0 a	60	2.0 ± 0.0 b	59	2.0 ± 0.0 b
3rd instar	80	2.0 ± 0.0 b	100	2.1 ± 0.0 a	60	2.1 ± 0.0 a	59	2.0 ± 0.0 b
4th instar	80	1.5 ± 0.1 c	100	2.2 ± 0.0 a	60	2.0 ± 0.0 b	59	2.0 ± 0.0 b
5th instar	78	3.0 ± 0.2 a	95	3.0 ± 0.1 a	60	2.7 ± 0.1 b	58	2.1 ± 0.0 c
6th instar	57	4.6 ± 0.1 bc	80	5.1 ± 0.2 ab	56	5.3 ± 0.2 a	56	4.5 ± 0.2 c
7th instar	/	/	18	5.9 ± 0.3 a	13	4.4 ± 0.3 b	10	3.9 ± 0.3 b
8th instar	/	/	1	6.0	/	/	/	/
Pupae	59	8.6 ± 0.1 b	63	9.7 ± 0.1 a	41	8.6 ± 0.1 b	54	8.6 ± 0.1 b
Female adult longevity (d)	27	12.6 ± 0.7 a	31	13.0 ± 0.8 a	19	7.9 ± 0.7 b	28	8.8 ± 0.5 b
Male adult longevity (d)	32	14.4 ± 0.9 a	32	10.4 ± 0.6 b	22	7.4 ± 0.9 c	26	7.5 ± 0.4 c
Total longevity (d)	80	35.9 ± 0.8 a	100	34.7 ± 1.2 ab	60	32.4 ± 0.9 b	60	33.2 ± 0.8 b
Proportion of female adults (*N_f_*/*N*) (%)	80	33.8 ± 5.3 b	100	31.0 ± 4.6 b	60	31.7 ± 6.0 b	60	47.7 ± 6.4 a
Proportion of fertile female adults (*N_fr_*/*N_f_*) (%)	80	96.3 ± 3.7 a	100	87.1 ± 6.1 ab	60	84.2 ± 8.5 ab	60	78.6 ± 7.8 b
Proportion of male adults (*N_m_*/*N*) (%)	80	40.0 ± 5.5 a	100	32.0 ± 4.7 a	60	36.7 ± 6.2 a	60	43.3 ± 6.4 a
APOP (d)	26	3.4 ± 0.2 b	27	5.7 ± 0.5 a	16	3.6 ± 0.3 b	22	3.7 ± 0.3 b
TPOP (d)	26	28.4 ± 0.4 d	27	35.9 ± 0.5 a	16	31.5 ± 0.3 b	22	29.7 ± 0.3 c
Oviposition day (*O_d_*) (d)	26	5.5 ± 0.2 a	27	4.4 ± 0.4 b	16	3.3 ± 0.2 c	22	3.7 ± 0.2 bc
Fecundity (eggs/female)	27	1054.6 ± 66.6 a	31	445.8 ± 62.4 c	19	439.7 ± 56.6 c	28	721.1 ± 84.1 b

Data are presented as mean ± SE. Different letters within each row indicate significant differences between treatments at the level of *α* = 0.05 (paired bootstrap test). APOP and TPOP refer to adult pre-oviposition period and total pre-oviposition period, respectively.

**Table 3:** Effects of continuous rearing on the leaves of Job’s tears cultivar ‘Cuiyi 1’ on the population parameters of FAW.

Population Parameter	Corn	Job’s Tears Cultivar ‘Cuiyi 1’		
	F2 Generation	F2 Generation	F5 Generation	F8 Generation
Intrinsic rate of increase, *r* (d^−1^)	0.193 ± 0.006 a	0.137 ± 0.006 c	0.138 ± 0.012 c	0.172 ± 0.008 b
Finite rate of increase, *λ* (d^−1^)	1.212 ± 0.007 a	1.147 ± 0.006 c	1.148 ± 0.014 c	1.188 ± 0.009 b
Net reproductive rate, *R*_0_	355.9 ± 60.0 a	179.0 ± 34.3 bc	92.7 ± 30.2 c	269.3 ± 60.5 ab
Mean generation time, *T* (d)	30.6 ± 0.3 c	37.8 ± 0.3 a	32.9 ± 0.6 b	32.6 ± 0.4 b

Data are presented as mean ± SE. Different letters within each row indicate significant differences between treatments at the level of *α* = 0.05 (paired bootstrap test).

**Table 4:** Effects of continuous rearing on the leaves of Job’s tears cultivar ‘Puiyi 6’ on the population parameters of FAW.

Population Parameter	Corn	Job’s Tears Cultivar ‘Puyi 6’		
	F2 Generation	F2 Generation	F5 Generation	F8 Generation
Intrinsic rate of increase, *r* (d^−1^)	0.193 ± 0.006 a	0.131 ± 0.006 b	0.148 ± 0.007 b	0.185 ± 0.006 a
Finite rate of increase, *λ* (d^−1^)	1.212 ± 0.007 a	1.109 ± 0.020 b	1.165 ± 0.010 b	1.185 ± 0.008 a
Net reproductive rate, *R*_0_	355.9 ± 60.0 a	138.2 ± 28.2 b	139.2 ± 31.7 b	336.5 ± 60.5 a
Mean generation time, *T* (d)	30.6 ± 0.3 d	37.6 ± 0.3 a	33.1 ± 0.4 b	31.4 ± 0.2 c

Data are presented as mean ± SE. Different letters within each row indicate significant differences between treatments at the level of *α* = 0.05 (paired bootstrap test).

## Data Availability

The original contributions presented in the study are included in the article/Supplementary Materials, further inquiries can be directed to the corresponding authors.

## Supplementary Materials

The following supporting information can be downloaded at: https://www.mdpi.com/article/10.3390/insects16080773/s1, Figure S1: Stage-size projection of *S. frugiperda* fed on corn for two generations (A) and continuous rearing on Job’s tears cultivars ‘Cuiyi 1’ (B–D) and ‘Puyi 6’ (E–G) for eight generations (F2: B, E; F5: C, F; F8: D, G); Table S1: Effects of continuous rearing on the leaves of Job’s tears cultivar ‘Cuyi 1’ on the survival of *Spodoptera frugiperda*; Table S2: Effects of continuous rearing on the leaves of Job’s tears cultivar ‘Puyi 6’ on the survival of *Spodoptera frugiperda*.
