# Supplementary material for: Effects of Multi-Generational Rearing on Job’s Tears on the Performance and Host Plant Preference of Spodoptera frugiperda (Lepidoptera: Noctuidae)
Source: Insects. 2025 Jul 28;16(8):773. doi: 10.3390/insects16080773 (PMC12386935; doi:10.3390/insects16080773)
Supplement: Supplementary file 1 [file insects-16-00773-s001.zip › insects-3706272-supplementary.pdf]

Supplementary Materials

Equations and definitions of life table performance parameters and population parameters

Life table performance parameters include age-stage specific survival rate (s_{xj}), age-specific survival rate (l_x), female age-stage specific fecundity (f_{xj}), age-specific fecundity (m_x), net maternity ($l_x m_x$), age-stage life expectancy (e_{xj}), and age-stage reproductive value (v_{xj}). Population parameters include net reproductive rate (R_0), intrinsic rate of increase (r), finite rate of increase (λ), and mean generation time (T).

The s_{xj} (x = age, j = stage) is the probability that a newborn will survive to age x and stage j, which is calculated as follows:

$$s_{xj} = \frac{n_{xj}}{n_{01}}$$

where n_{01} is the number of individuals used at the beginning of the life table study, and n_{xj} is the number of individuals surviving to age x and stage j.

The l_x is the probability that a newborn will survive to age x, which is calculated as follows:

$$l_x = \sum_{j=1}^{\beta} s_{xj}$$

where β is the number of stages.

The m_x is the average number of eggs produced by an individual at age x, which is calculated as follows:

$$m_x = \frac{\sum_{j=1}^{\beta} S_{xj} f_{xj}}{\sum_{j=1}^{\beta} S_{xj}}$$

where fecundity f_{xj} the daily number of eggs laid by a female adult at age x and stage j.

The R_0 is defined as the total number of offspring that an individual can produce during its lifetime, which is calculated as follows:

$$R_0 = \sum_{x=0}^{\infty} l_x m_x$$

The r is the population growth rate as time approaches infinity and population reaches the stable age-stage distribution. The population size will increase at the rate of e^r or λ per time unit. The r is calculated using the Lotka–Euler equation with age indexed from 0 as follows:

$$\sum_{x=0}^{\infty} e^{-r(x+1)} l_x m_x = 1$$

The λ is calculated as follows:

$$\lambda = e^r$$

The T represents the period that a population requires to increase to R_0-fold of its size as time approaches infinity and the population settles down to a stable age-stage distribution, which is calculated as follows:

$$T = \frac{\ln R_0}{r}$$

The e_{xj} is the duration that an individual of age x and stage j is expected to survive after age x, and is calculated as follows:

$$e_{xj} = \sum_{i=x}^{\infty} \sum_{y=j}^{\beta} s'_{iy}$$

where s'_{iy} is the probability that an individual of age x and stage j will survive to age i and stage y, and it is calculated by assuming s'_{iy} = 1.

The v_{xj} is the contribution of individuals of age x and stage y to the future population. It is calculated as follows:

$$v_{xj} = \frac{e^{r(x+1)}}{s_{xj}} \sum_{i=x}^{\infty} e^{-r(i+1)} \sum_{y=j}^{\beta} s'_{iy} f_{iy}$$

Table S1. Effects of continuous rearing on the leaves of Job's tears cultivar 'Cuyi 1' on the survival of *Spodoptera frugiperda.*

Parameters	Corn		Job's tears cultivar 'Cuiyi 1'					
	n	F2 generation	*n*	F2 generation	*n*	F5 generation	*n*	F8 generation
Survival rate to the succeeding stage (%)								
Pre-adult	80	73.8 ± 4.9 a	100	68.0 ± 4.7 a	60	60.0 ± 6.3 a	60	75.0 ± 5.6 a
Eggs	80	100 a	100	100 a	60	100 a	60	100 a
Larvae	80	96.3 ± 2.1 a	70	70.0 ± 4.6 b	49	81.7 ± 5.0 b	48	80.0 ± 5.2 b
1st instar	80	100 a	100	85.0 ± 3.6 b	60	100 a	60	85.0 ± 4.6 b
2nd instar	80	100 a	85	96.5 ± 2.0 a	60	100 a	51	98.0 ± 1.9 a
3rd instar	80	100 a	82	98.8 ± 1.2 a	60	98.3 ± 1.7 a	50	100 a
4th instar	80	100 a	81	98.8 ± 1.2 a	59	98.3 ± 1.7 a	50	100 a
5th instar	80	97.5 ± 1.7 a	80	100 a	58	98.3 ± 1.7 a	50	100 a
6th instar	58	98.3 ± 1.7 a	80	97.5 ± 1.7 ab	57	89.5 ± 4.1 b	50	98.0 ± 2.0 ab
7th instar	/	/	62	87.1 ± 4.3 a	19	89.5 ± 7.1 a	13	92.3 ± 7.4 a
8th instar	/	/	7	100 a	1	100 a	/	/
Pupae	77	76.6 ± 4.8 b	70	97.1 ± 2.0 a	49	73.5 ± 6.3 b	48	93.8 ± 3.5 a

Data in the table are shown as mean ± SE. Different letters in each row indicate significant differences between treatments at the level of $\alpha = 0.05$ (paired bootstrap test).

Table S2. Effects of continuous rearing on the leaves of Job's tears cultivar 'Puyi 6' on the survival of *Spodoptera frugiperda.*

Parameters	Corn		Job's tears cultivar 'Puiyi 6'					
	n	F2 generation	*n*	F2 generation	*n*	F5 generation	*n*	F8 generation
Survival rate to the succeeding stage (%)								
Pre-adult	80	73.8 ± 4.9 b	100	63.0 ± 4.8 b	60	68.3 ± 6.0 b	60	90.0 ± 3.9 a
Eggs	80	100 a	100	100 a	60	100 a	60	100 a
Larvae	80	96.3 ± 2.1 a	100	67.0 ± 4.7 c	60	86.7 ± 4.4 b	60	93.3 ± 3.2 ab
1st instar	80	100 a	100	100 a	60	100 a	60	98.3 ± 1.6 a
2nd instar	80	100 a	100	100 a	60	100 a	59	100 a
3rd instar	80	100 a	100	100 a	60	100 a	59	100 a
4th instar	80	100 a	100	100 a	60	100 a	59	100 a
5th instar	80	97.5 ± 1.7 ab	100	95.0 ± 2.2 b	60	100 a	59	98.3 ± 1.7 ab
6th instar	58	98.3 ± 1.7 a	95	84.2 ± 3.7 b	60	93.3 ± 3.2 ab	58	96.6 ± 2.4 a
7th instar	/	/	31	58.1 ± 8.9 b	17	76.5 ± 10.3 b	10	100 a
8th instar	/	/	1	100	/	/	/	/
Pupae	77	76.6 ± 4.8 b	67	94.0 ± 2.9 a	52	78.8 ± 5.7 b	56	96.4 ± 2.5 a

Data in the table are shown as mean ± SE. Different letters in each row indicate significant differences between treatments at the level of $\alpha = 0.05$ (paired bootstrap test).

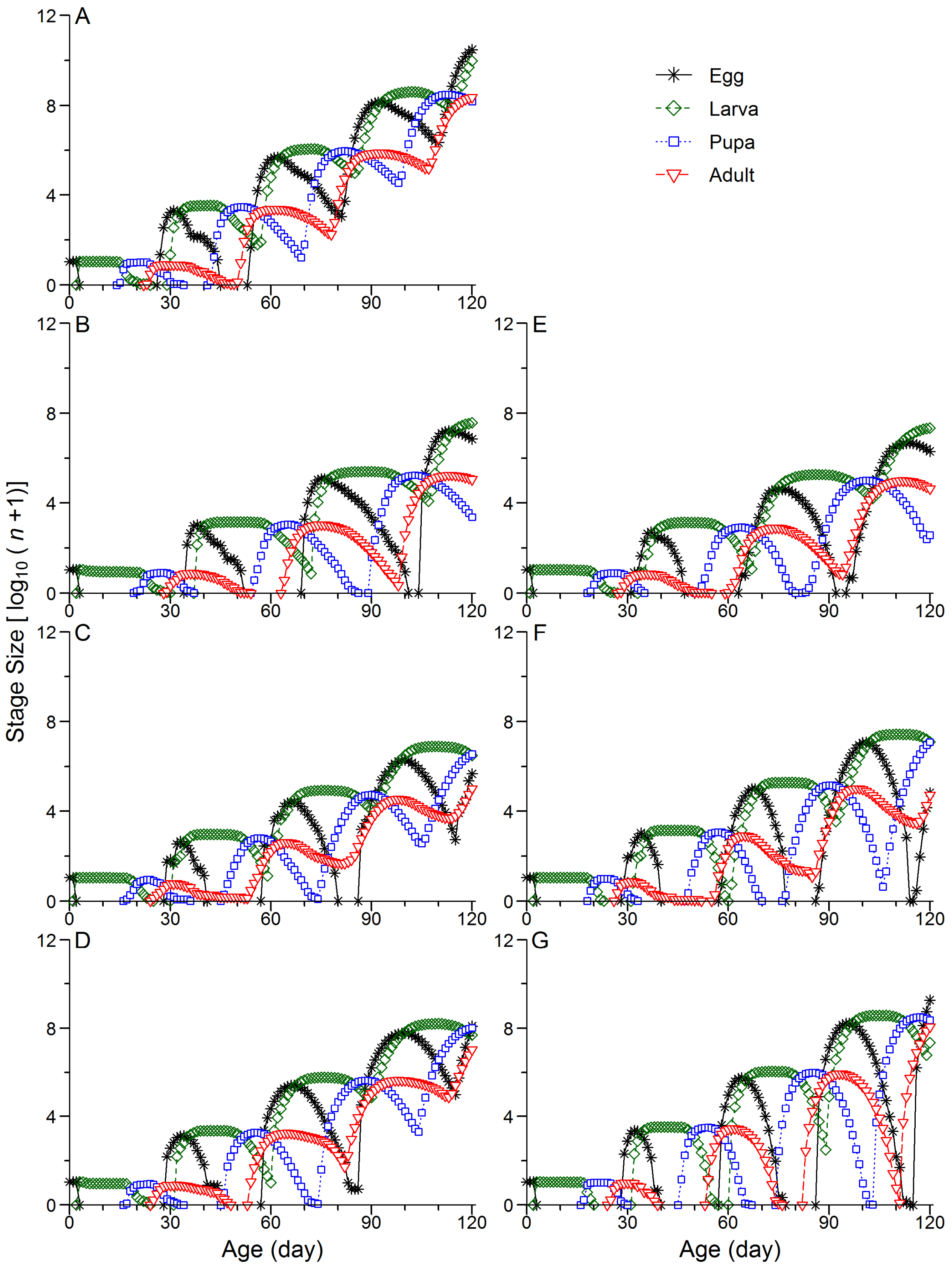

Figure S1. Stage-size projection of *S. frugiperda* fed on corn for two generations (A) and continuous rearing on Job's tears cultivars 'Cuiyi 1' (B – D) and 'Puyi 6' (E – G) for eight generations (F2: B, E; F5: C, F; F8: D, G).
